# Passing the Wake: Using Multiple Fins to Shape Forces for Swimming

**DOI:** 10.3390/biomimetics4010023

**Published:** 2019-03-12

**Authors:** Anthony P. Mignano, Shraman Kadapa, James L. Tangorra, George V. Lauder

**Affiliations:** 1 Department of Mechanical Engineering, College of Engineering, Drexel University, Philadelphia, PA 19104, USA; shraman.kadapa@drexel.edu (S.K.); tangorra@coe.drexel.edu (J.L.T.); 2 Department of Organismic and Evolutionary Biology, Harvard University, Cambridge, MA 02138, USA; glauder@oeb.harvard.edu

**Keywords:** fish, fin–fin interaction, biorobotic, multifin, computational fluid dynamics, flow visualization

## Abstract

Fish use coordinated motions of multiple fins and their body to swim and maneuver underwater with more agility than contemporary unmanned underwater vehicles (UUVs). The location, utilization and kinematics of fins vary for different locomotory tasks and fish species. The relative position and timing (phase) of fins affects how the downstream fins interact with the wake shed by the upstream fins and body, and change the magnitude and temporal profile of the net force vector. A multifin biorobotic experimental platform and a two-dimensional computational fluid dynamic simulation were used to understand how the propulsive forces produced by multiple fins were affected by the phase and geometric relationships between them. This investigation has revealed that forces produced by interacting fins are very different from the vector sum of forces from combinations of noninteracting fins, and that manipulating the phase and location of multiple interacting fins greatly affect the magnitude and shape of the produced propulsive forces. The changes in net forces are due, in large part, to time-varying wakes from dorsal and anal fins altering the flow experienced by the downstream body and caudal fin. These findings represent a potentially powerful means of manipulating the swimming forces produced by multifinned robotic systems.

## 1. Introduction

Fish swim and maneuver underwater with more agility than contemporary unmanned underwater vehicles (UUVs), and unlike the predominately rectilinear motions of UUVs, fish continually pitch, roll, turn and dynamically position their body in flow. They achieve this with coordinated motions of their body and fins, and the forces produced by each fin vary in magnitude and direction during each fin beat [1,2]. Rarely are the forces that are required for propulsion and stability created by a single fin or by fins that do not interact with wakes shed by the body, other fins, or finlets [1,3,4,5]. This wake interaction is affected by many factors and is likely dependent on the kinematic coordination between the fins, and how the wake of an upstream fin travels along the body. Fin location, size, utilization and kinematics vary for different species of fish [5,6]. Consequently, fin coordination varies due to fin motion being tied to body geometry and the undulating body wave as fish swim [7,8,9,10]. Fish vary the utilization and kinematics of their fins and body depending on swimming speed and/or locomotory task [3,11,12,13]. Although distinct gait changes in fish have been observed and are recognized to change the magnitude and direction of fin forces, variation in the phase, frequency and amplitude of fins can also be seen as speed changes [4,14,15,16]. During steady swimming, the caudal fin encounters the time-varying wake shed by the upstream fins and body [5,7,17,18]. The relative position and timing (phase) of the fins affects how the downstream fins interact with the wake shed by the upstream fins and body.

Despite many investigations of the forces produced by single fins, and several instances of multifinned underwater robots, the effect of coordination and relative location of fins on the forces is not well understood [1,2,19,20]. To date, much of the research on the flow interactions of individual appendages has been on insect wings during flight [21,22,23,24]. In two-dimensional (2D) numerical simulations in air, for wings that interact with the vortex produced by upstream wings, the lift and thrust coefficients, as well as efficiency are affected by the relative phase angle and distance between wings [25,26]. In the underwater domain, forces produced by single fins are frequently investigated and have been included in the design of many underwater robots [27,28,29,30]. There is also computational fluid dynamic evidence that flow shed from rigid fins and finlets impacts the downstream fin forces and may improve their efficiency and thrust coefficient [31,32]. Experimental research on live fishes has considered the mechanics of propulsion with multiple fins, and some studies have characterized fin kinematics of fish using multiple fins during steady swimming and infer that asynchronous flapping of fins may help stabilize the fish body and produce relatively constant thrust [6,33,34]. Furthermore, several multifinned swimming robots have been produced, some highlighting the propulsive advantages of using multiple fins [35,36]. Flammang et al. [18] have shown, using three-dimensional (3D) volumetric flow visualization with live fish, that the vortex wake being shed by the dorsal and anal fins was entrained by the tail within a single fin beat. However, only a few studies have discussed how varying phase and relative body position of fins with complex 3D kinematics affect the net forces [37].

The objective of this investigation is to understand how the propulsive forces produced by multiple fins are affected by the phase and geometric relationships between them. To that end, the remainder of this paper will discuss: (1) the development and experimental use of a multifin biorobotic experimental platform with variable phase and geometric relationships between the median fins and peduncle; (2) a 2D numerical hydrodynamic simulation of two rigid flapping foils to visualize the flow interactions and estimate propulsive forces of interacting fins; (3) the effect of fin phase and geometric relationships on the mean and time-varying shape of propulsive forces and the associated experimental and simulated flows. 

## 2. Materials and Methods

### 2.1. Biorobotic Studies

**Figure 1:**
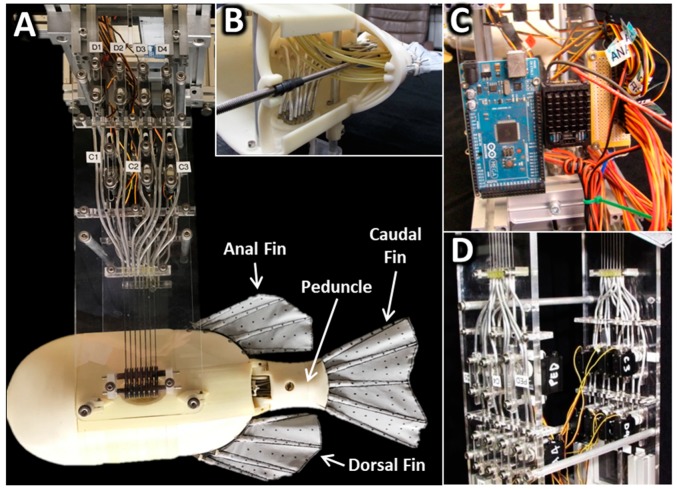
(**A**) The peduncle, dorsal, anal and caudal fin (PDAC) robot and support structure. (**B**) The position of the dorsal fin can be adjusted by turning a threaded rod. (**C**) Servomotors are controlled using a microcontroller. (**D**) Fin rays are driven by servos via tendons that travel through reinforced nylon conduit.

A multifin robotic platform was developed and used to experimentally evaluate how the location and phase relationships between the peduncle (the narrow region of fish just in front of the tail) and the dorsal, anal and caudal fins (referred to as PDAC) affect thrust, lateral forces, and wakes during steady swimming (Figure 1). The PDAC robot is comprised of three systems: (1) a fish-shaped body with an actuated peduncle; (2) three fins (caudal, dorsal and anal), each with individually actuated fin rays; and (3) a support structure that holds the robot for testing in flow and on which the actuators and electronics used to drive the robot are mounted.

#### 2.1.1. PDAC Body

The body of the PDAC robot is a fish-shaped, acrylonitrile butadiene styrene (ABS) shell. The body shape and fin locations were derived from computed tomography (CT) scans and high-resolution images of the bluegill sunfish (*Lepomis machrochirus*) and rainbow trout (*Oncorhynchus mykiss*). The scans and images were used to create a parametrized 3D model of the robot body (CREO Parametric 3.0, PTC, Needham, MA, USA). The parametrization allowed the 3D model to be scaled to fit internal mechanical systems and a size appropriate for our testing environment (Table 1). The 3D model was divided into several sections for ease of manufacture and assembly. All body sections were fabricated from ABS via fused deposition modeling (FDM, Dimension Elite, Stratasys Ltd., Eden Prairie, MN, USA). 

**Figure 2:**
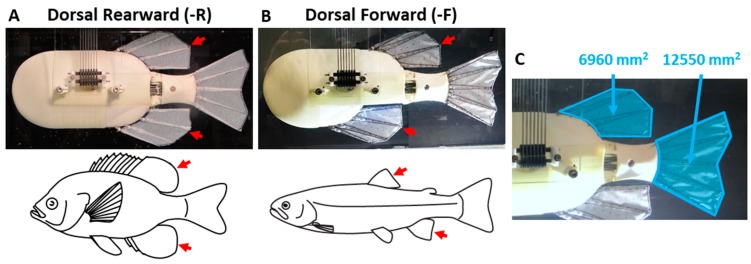
(**A**) PDAC robot in the dorsal-rearward and (**B**) dorsal-forward configurations, corresponding to the median fin geometries of the sunfish and trout, respectively. Experiments were conducted with the robot positioned upside-down to allow the body, dorsal and caudal fins to be filmed through the transparent bottom of the tank. (**C**) The dorsal and anal fins are shaped identically and each of them are 55% of the area of the caudal fin.

The body was designed so that the dorsal fin could be moved rostrocaudally and different fin configurations could be tested (Figure 2). The dorsal fin is attached to a threaded rod that, when rotated, moves the dorsal fin forward or aft along the body (Figure 1B). Rigid spacers are used to fill the gaps in the body as the dorsal fin is re-positioned. Two dorsal fin configurations were tested; a dorsal rearward configuration (-R) where the dorsal and anal fins are in a symmetrical arrangement (Figure 2A), and a dorsal-forward (-F) configuration in which the dorsal fin is positioned 82 mm closer to the front of the body (Figure 2B). These dorsal fin positions correspond approximately to the dorsal and anal fin positions of the sunfish (symmetric) and trout (asymmetric). The peduncle is located just posterior of the dorsal and anal fins and connects the body to the caudal fin. The peduncle is attached via a revolute joint and is designed to be easily replaced with peduncles of different lengths to vary the distance between the caudal fin and the rest of the body and fins. The peduncle length used in this investigation corresponds to the scaled average length of the peduncles of the model organisms. 

#### 2.1.2. Fins

Each of the fins (dorsal, anal and caudal) consist of multiple, individually actuated fin rays covered in a 84% polyester/16% elastane webbing (Under Armour, Inc., Baltimore, MD, USA) (Figure 2C). The geometry of each robotic fin was chosen to allow the fins to approximate selected gaits (steady swimming, backing, and turns) while using a minimal number of fin rays within each fin to produce the desired fin shape while flapping. Kinematics and dominant changes in fin shape were identified from observations of biological fish during a variety of gaits [6,27,38]. It was determined that four fin rays for each of the dorsal and anal fins and five fin rays for the caudal fin were necessary to capture much of the observed biological fin motion [29,34,39,40]. Although the other fin rays of the biological fins add to each fin’s structure during a motion, they are generally located in areas where the shape was bounded by the fin rays selected for each fin, and are omitted to simplify the design of the robot. Each fin ray is attached to a hinge on a curved base with the fin rays in the sagittal plane. This curved base causes each fin to cup along its spanwise axis when flapped which mimics the cupping observed during locomotion in fishes [5,6,27,38].

The dimensions of each robotic fin ray were tuned such that their bending was consistent with the fin bending observed during steady swimming in vivo in previous 3D kinematic studies [3,5,6,27]. The length of each fin ray was scaled to match the PDAC robot body size; with the longest fin rays in the caudal fin (110 mm) and the shortest being the medial most fin rays of the dorsal/anal fin (85 mm). The width and height of each fin ray tapers along their lengths, such that they are stiffest at their base and most flexible near their tip [29,41]. Each fin ray was manufactured from ABS using fused deposition modeling, in the same process as the robot body.

#### 2.1.3. Support Structure

The support structure suspends the PDAC body and fins inside a flow tank for testing while also serving as mounting points for a microcontroller, actuators and a series of shrouded tendons that attach the actuators to fin rays. The support structure is made up of two acrylic panels, held parallel by six 10 mm diameter aluminum rods (Figure 1A). Two aluminum rods at the bottom of the support structure pass through and attach to the PDAC body. An extruded aluminum frame at the top of the support structure attaches the PDAC robot to the flow tank. Also at the top, inside the frame, are the microcontroller and power distribution circuitry that control and power the banks of actuators mounted on the inside of each acrylic panel (Figure 1C). 

The actuators mounted on the support structure drive the fins and peduncle through a tendon-conduit mechanism that allows the fins to be quickly repositioned independently of the actuators’ locations (Figure 1D). Each fin ray and the peduncle are actuated by a rotational servomotor (Hitec RCD USA, Inc., Poway, CA, USA) via low-stretch, nylon-coated, stainless steel wire rope, acting as tendons in a pull–pull configuration (McMaster-Carr, No. 34235T26). The tendons are routed such that they always maintain a fixed length to their associated fin ray. From the servomotors, each tendon is routed vertically along the acrylic panels, through aluminum-reinforced nylon tubing until it reaches a manifold (Figure 1D). At the manifold, the conduits are arranged to condense all the tendons from the banks of servomotors into two, tightly aligned rows (Figure 1D). The tendons then pass through another manifold on each side of the body and are routed to the base of each fin ray (Figure 1B). By locating the actuators outside the body and above water, the tendon-conduit mechanism allows for more flexibility in the design of the body and fins to test a wider range of body geometries.

The servomotors are driven by a microcontroller which has been programmed to output the necessary pulse-width modulation signals (ATmega2560, Atmel Corp., San Jose, CA, USA). The microcontroller receives the desired fin ray motion trajectories from an attached personal computer (PC). The motion trajectories were derived from our previous biological and fish robotic research [1,3,6,29,36], and tuned for the robot using MATLAB (MathWorks Inc., Natick, MA, USA).

#### 2.1.4. Testing Environment

To measure thrust and lateral forces, the PDAC robot was mounted on a carriage riding on low-friction, precision air bushings (New Way Air Bearings, Aston, PA, USA), and suspended in a recirculating flow tank. Forces were measured using two load cells (4.45N, LSB200, Futek Inc., Irvine, CA, USA) and sampled at 250 Hz using a National Instruments USB-6229 data acquisition board (National Instruments Corp., Austin, TX, USA) as in our previous research [29,36,41]. 

#### 2.1.5. Experimental Methods

The robot was tested in a flow tank so that propulsive forces and flows could be measured at steady swimming speeds. The robot was positioned upside-down to allow the flow surrounding the body, dorsal and caudal fins to be observed through the transparent bottom of the tank (Figure 2A). Two high-speed cameras (Photron FASTCAM, Photron USA, Inc., San Diego, CA, USA) were positioned to capture the lateral and dorsal views of the robot simultaneously with the force measurements. Raw videos were captured at 500 fps at a resolution of 1024 × 1024 pixels. Hydrodynamic wakes produced by the PDAC robot were studied using digital particle image velocimetry (DPIV), as in previous research [5,6,29,34,41,42]. The laser-excited light sheet was projected orthogonal to the plane of the caudal fin and aligned with the region where the dorsal shed wake would interact with the caudal fin (Figure 2A,B). Captured images of the generated wake flow patterns were compared to in vivo wake flows from previous experiments [5,6,38].

All experiments were conducted at a flow speed of 200 mm/s, which corresponds to 0.43 body lengths per second (BL/s). This flow speed corresponds to a self-propelling speed of the PDAC robot with the caudal fin and peduncle (60° phase lead) flapping at 1 Hz while keeping the dorsal and anal fins stationary and aligned with the body. Under these conditions, the flow surrounding the PDAC robot as it is tested is consistent with those found in vivo, and corresponds to a Strouhal number of approximately 0.5 and a Reynolds number of 105,600 [43]. Furthermore, investigations of flapping foils by Van Buren et al. [44] suggest that measurements of fin forces and flows using the PDAC robot in this way will not differ significantly from those produced by a free-swimming PDAC robot.

Experiments were conducted (*n* = 127) to determine how variations in the relative phase and distance between the dorsal, anal and caudal fins affect the magnitude and time-varying shape of the produced thrust and lateral forces. Experiments were divided into six sets, covering three fin combinations at each of two geometric configurations of the body. For each fin combination, fins that were not used were held stationary and aligned with the body for the duration of the trial. The fins that were flapped for each of the three combinations tested were: dorsal and caudal fins only (DC) (*n* = 75); dorsal, anal and caudal fins (DAC) (*n* = 34); peduncle, dorsal, anal and caudal fins (PDAC) (*n* = 18). The two body geometry configurations tested were the rearward (-R) configuration (*n* = 72), where the dorsal fin was symmetric with the anal fin (Figure 2A) and the forward (-F) configuration (*n* = 55), where the dorsal fin was 82 mm ahead of the anal fin (Figure 2B). 

When not stationary, fins and the peduncle were flapped at 1 Hz. The flapping amplitudes of the fin rays in each of the dorsal and anal fins ranged from 10° to 17°, and were selected so that each fin created a cupping motion along the spanwise axis when flapped, which has been shown to be beneficial in the production of thrust forces [29]. Caudal fin ray amplitude ranged from 25° for the outermost fin rays to 14° for the middle fin ray. The peduncle, when not stationary, was flapped with an amplitude of 22°. To establish baselines of fin forces and help build a model of forces, the forces produced by the individual dorsal, anal, and caudal fins were measured for both the -R and -F configurations.

For each set of experiments, the phase of the dorsal/anal fins was varied relative to the caudal fin. During the DC and DAC experiment sets, the dorsal/anal fin phase was varied from −180° to 180° relative to the caudal fin. During the PDAC experiment sets, the dorsal/anal fin phase was varied, but the peduncle was flapped in-phase with the caudal fin. 

#### 2.1.6. Experimental Validation

To verify that the experimental results were broadly representative of forces created by interacting fins, validation tests were conducted across an extensive set of conditions. Experiments were also conducted with the PDAC robot at flow speeds above and below the reported self-propulsion speed (150 and 250 mm/s; 0.32 and 0.53 BL/s_,_ respectively; *n* = 57), in static water (*n* = 44), with different flapping frequencies (0.75 and 1.25 Hz; *n* = 27 and 29, respectively), and with the dorsal and anal fins positioned at different locations relative to the body along the dorsoventral axis (*n* = 54). Experiments were also conducted to track the body motion (surge, sway and yaw) of a free-swimming robot while executing fin kinematics similar to those of the PDAC robot, except for the peduncle actuation (*n* = 18) [45]. 

#### 2.1.7. Data Processing

For each experimental trial, 10 fin beat cycles of recorded thrust and lateral forces were processed to produce force traces and averages for further analysis. The raw thrust and lateral forces were processed with a median filter followed by a low-pass filter. The median filter used a five-sample window and the low-pass filter used a Kaiser window with a passband frequency of 9 Hz, a stopband frequency of 12 Hz and a peak error of 10^−3^ [46]. To minimize the inclusion of transients associated with the beginning of fin motion, recorded forces from the first three fin beat cycles were discarded. Forces from the subsequent 10 cycles in each trial were synchronized using a timing signal generated by the microcontroller. Mean net forces and standard deviations (SD) were then calculated from these force recordings. Since the mean of the lateral force over each fin beat was approximately zero, the root mean square (RMS) rather than the mean is used to quantify the net lateral forces. 

To characterize changes in the time-varying shape of forces, the magnitudes of peak thrust and drag, and the relative durations of thrust or drag producing portions of the fin beats were calculated. Magnitudes of peak thrust and drag were found for each of the fin beat cycles and used to calculate mean values. The duration of the fin beat that produces thrust, as a fraction of the total fin beat, was calculated by finding the time within each fin beat during which thrust was produced and dividing that by the total duration of the fin beat. Similar calculations were used to determine the relative duration of the drag producing portions of the fin beat.

### 2.2. Computational Fluid Dynamics Simulation

#### 2.2.1. Geometric Modeling

**Figure 3:**
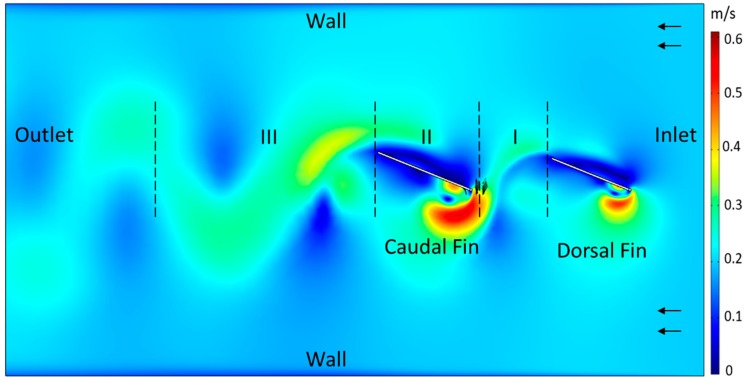
Geometric setup of numerical simulations of 2D flapping dorsal and caudal fins. Dorsal fin wake (I); flow around caudal fin (II); caudal fin wake (III).

To help visualize flow as the relative phase between two interacting rigid fins is varied, numerical 2D simulations were developed (COMSOL Multiphysics, COMSOL, Inc., Boston, MA, USA) (Figure 3). The dorsal fin and the caudal fin were flapped inside a rectangular shaped tank which is 750 mm long and 400 mm wide. The size and location of each fin was chosen to approximately match those of the PDAC robot fins captured in the laser-light sheet during DPIV experiments. The body of PDAC robot was not included in the simulation as it does not intersect with the flow in the region of interest. The 2D rectangular fins were 90 mm and 107.5 mm long, respectively, and 2 mm wide with infinite depth. The caudal fin was placed directly behind the dorsal fin so that it interacted with the wake shed by the dorsal fin (Figure 3). The distance between the trailing edge of the dorsal fin and the leading edge of the caudal fin was 80 mm. Fluid flow was directed parallel to the rigid fins. The force component that is perpendicular to the inlet flow direction is lateral force. The force component that is parallel to the inlet flow direction is thrust force. The projections of line integral of the total stress on all the boundaries of the fins were used to compute the thrust and lateral forces as the fins were flapped.

#### 2.2.2. Numerical Simulations

Numerical simulations were conducted to visualize flow and calculate forces as the relative phase difference between the fins was varied (Figure 3). The turbulent k-Ɛ model with Reynolds-averaged Navier–Stokes (RANS) formulation was used for the model [47]. The dorsal fin and the caudal fin were flapped in the rectangular 2D tank with a simple sinusoidal trajectory with a frequency of 1 Hz and amplitude of ±22° in a 200 mm/s inflow (0.43 BL/s). Simulations were conducted at the following phase difference values: −180°, −165°, −160°, −135°, −105°, −90°, −45°, −20°, −10°, 0°, 20°, 45°, 90°, 135°, 158°, 170°, and 180°. The slight inconsistency in the phase difference values was due to the way the phase was determined by the COMSOL software. To better understand flow dynamics, the simulated environment was examined at three different locations: the dorsal fin wake, flow around the caudal fin, and the wake shed by caudal fin (Figure 3).

#### 2.2.3. Data Processing

Numerically computed thrust and lateral forces were processed to analyze wake dynamics in the simulated tank. To minimize the effect of transience in the system, the data from the first three fin cycles was ignored. The forces from five fin beats was used to calculate the mean thrust and RMS lateral forces for each simulated fin phase. Due to the 2D plane strain assumption, it is assumed that the force distribution along the out of plane direction is the same. The resulting thrust and lateral forces were multiplied by 125 mm which corresponds to the assumed height of both fins. The angle of attack of the caudal fin was measured for three fin beats and the values were averaged to determine how it varied as relative phase between the fins was changed. 

## 3. Results

Experimentation conducted with the biorobotic experimental platform shows that changes in the relative phase of the fins significantly impact the magnitude and temporal profile of the net force vector. Furthermore, variations in the geometric relationship among the fins affect the phase at which the changes in forces occur. Observations of experimentally recorded and simulated flows suggest that these changes in forces may be attributed, in large part, to the interaction of the downstream fins and body with the wake produced by the upstream fin(s).

### 3.1. Forces from Single Fins (Baseline)

**Figure 4:**
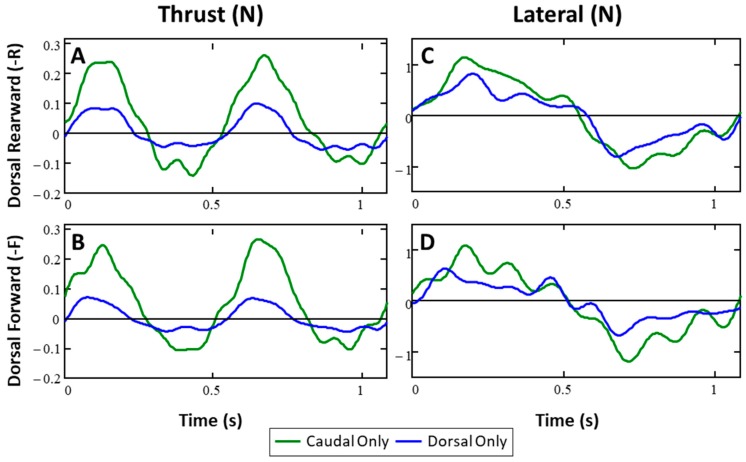
(**A**,**B**) Thrust forces for dorsal-rearward and dorsal-forward configurations, respectively. (**C**,**D**) Lateral forces for dorsal-rearward and dorsal-forward configurations, respectively, from caudal and dorsal fins operated alone are characteristic of flexible fin rayed fins.

The forces produced by the dorsal, anal and caudal fins—when each fin was operated alone— were typical of those expected for undulating, flexible fins [1,5,6,29]. Each fin produced thrust as it moved with the flow from its most lateral position toward and past the midline of the body and then produced drag as it moved into the flow toward its most lateral position to the opposite side. This thrust and drag pattern was repeated as the fin completed the second half of the fin beat and moved back to its starting position (Figure 4A,B). For the caudal fin, the magnitude and duration of the thrust peaks were greater than the magnitude and duration of the drag peaks, resulting in a net positive thrust. Over the entire fin beat, mean thrust for the caudal fin was 0.045N (SD = ±0.006 N, *n* = 14) when the dorsal fin was positioned symmetrically with the anal fin (-R) and was slightly higher, 0.050 N (SD = ±0.006 N, *n* = 16), when the dorsal fin was positioned forward (-F; Figure 4B). For the dorsal and anal fins, the thrust and drag peaks were nearly equal in magnitude and duration, resulting in approximately zero mean thrust over the course of the fin beat. This was true whether the dorsal fin was positioned forward or rearward. Lateral forces from the fins exhibited two opposing peaks per fin beat (Figure 4C,D) with peak magnitudes that were three or more times greater than the magnitudes of the thrust and drag peaks. However, since the lateral forces are directed in opposite directions for each of two halves of the fin beat, the net lateral force over the fin beat was approximately zero. The shape and magnitude of the lateral force from each fin were not affected significantly by the forward or rearward location of the dorsal fin. 

### 3.2. Mean Thrust and Root Mean Square Lateral Forces

**Figure 5:**
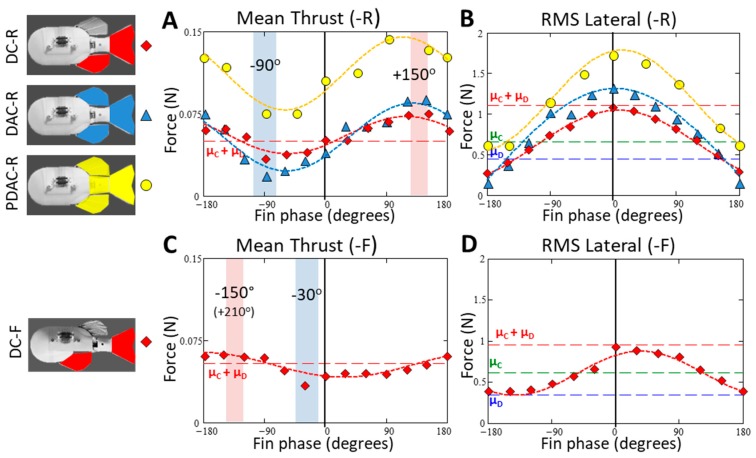
(**A**,**B**) Mean thrust and root mean square (RMS) lateral forces produced by different combinations of fins in the dorsal-rearward configuration, respectively. (**C**,**D**) Mean thrust and RMS lateral forces produced by the dorsal and caudal fins in dorsal-forward configuration, respectively. Recorded forces vary with respect to mean forces produced by dorsal and caudal fins when flapped alone (μ_C_ and μ_D_, respectively). When flapped, the peduncle is always in phase with the caudal fin.

For all four tested fin combinations/configurations (DC-R, DAC-R, PDAC-R and DC-F) mean thrust and RMS lateral forces varied cyclically as the phase of the dorsal and anal fin was varied relative to the phase of the caudal fin and peduncle (Figure 5). The changes in force as phase was varied were large—for example, for DAC-R, relative to the forces produced when the fins were flapped in phase, changing the phase of the dorsal and anal fins could increase mean thrust by over 90% or alternatively decrease thrust by over 50%, and lateral forces could be reduced by over 85%.

Though the magnitude of the mean thrust forces varied depending on the fin combination, the phase at which the maximum and minimum thrust forces were measured was the same for all combinations in the -R configuration. Changing the position of the dorsal fin from the -R to the -F configuration shifted the thrust-phase curve by approximately 60° (Figure 5).

Similarly, the magnitude of the maximum and minimum lateral forces varied depending on which combination of fins were flapped. However, in contrast to the thrust-phase curves, the RMS lateral force vs. phase curves shifted little when the position of the dorsal fin was changed. For all four of the tested fin combination/configurations, maximum lateral forces were produced when the fins were flapped in phase and minimum lateral forces were produced when the dorsal and anal fins were flapped at 180° phase relative to the caudal fin.

#### 3.2.1. Mean Thrust vs. Phase

Mean thrust varied smoothly, and was modeled well as a sinusoid curve, as the phase of the dorsal and anal fin was changed relative to the caudal fin (Figure 5), with a pattern that was similar for all fin combinations. The force–phase pattern shifted in phase when the dorsal fin was moved from the rearward (-R) to the forward position (-F). 

For all three fin combinations in the -R configuration, when the fins were flapped in phase (0°), the mean thrust from the interacting fins was approximately equal to the mean thrust predicted by summing mean forces from the individual fins (Figure 5). As the phase of the dorsal and anal fins was increased relative to the caudal fin and peduncle, the mean thrust increased monotonically until reaching a peak at a phase lead of approximately 150°. As the phase lead relative to the caudal fin was increased past 150°, mean thrust decreased steadily until it reached a minimum at approximately 270° (lag of 90°). Mean thrust then increased as the fins were brought back into phase (0°). 

Though a similar cyclical force–phase pattern was produced by each fin combination, the range of mean thrust differed relative to the baseline forces measured when each combination of fins was flapped in phase (Figure 5A, Table 2). Flapping the dorsal and caudal fins together (DC combination) produced 0.052 N (SD = ±0.004 N, *n* = 13) of thrust when the fins were flapped in phase, a minimum mean thrust of 0.035 N (SD = ±0.004 N, *n* = 13) at a phase lead of 270° (−90°), and a maximum mean thrust of 0.077 N (SD = ±0.007 N, *n* = 13) at 150°. For the DC combination, mean thrust ranged from a minimum of 0.7 (Δ = −0.016 N) to a maximum of 1.4 times (Δ = +0.025 N) the baseline thrust (0°). The addition of the anal fin (DAC combination) did not appreciably change the mean thrust produced when the fins were flapped in phase (0.039 N, SD = ±0.005 N, *n* = 14), but did affect the shape of the thrust curves (discussed in Section 3.3.3) and increased the range of mean forces produced across phases. Minimum and maximum mean thrust forces for the DAC combination were 0.020 N (SD = ±0.005 N, *n* = 17) and 0.087 N (SD = ±0.006 N, *n* = 13), respectively, which is a range of 0.5 (Δ = −0.019 N) to 2.2 times (Δ = +0.048 N) the baseline mean thrust produced when the fins were flapped in phase (0°). The inclusion of the anal fin increased the maximum mean thrust to 1.1 times and reduced minimum mean thrust to 0.6 times of the maximum and minimum thrust produced by the DC fin combination.

The inclusion of the flapping peduncle (PDAC) significantly increased the thrust produced at all phases (Figure 5A) relative to the DAC combination. However, the range between the minimum and maximum mean thrusts relative to the PDAC in-phase baseline was not augmented by the addition of the flapping peduncle, and was nearly the same as the DAC combination. The magnitude of PDAC baseline mean thrust (0°) was 0.108 N (SD = ±0.006 N, *n* = 18) and was 2.8 times larger than the fin only (DAC) thrust baseline. The minimum and maximum mean thrust produced with PDAC combination was 0.076 N (SD = ±0.007 N, *n* = 18) and 0.146 N (SD = ±0.005 N, *n* = 18), respectively, a range of 0.7 (Δ = −0.032 N) to 1.4 times (Δ = +0.038 N) the magnitude of the baseline PDAC thrust forces.

When the dorsal fin was moved to the forward position (-F), the general shape of the mean thrust vs. phase curve did not change, but the curve was shifted by approximately +60°, relative to the curve produced with the dorsal in the rearward position (-R; Figure 5C). Maximum mean thrust for the -F configuration occurred at a phase lead of 210° (−150°), compared to 150° for the -R configuration. Similarly, the minimum mean thrust for the -F configuration occurred at a phase lead of 330° (−30°), compared to 270° (−90°) for the -R configuration. The range of minimum and maximum mean thrust produced by the -F configuration was very similar to the -R configuration, producing 0.034 N (SD = ±0.005 N, *n* = 18) and 0.065 N (SD = ±0.003 N, *n* = 17), respectively, a range of 0.7 (Δ = −0.012 N) to 1.4 (Δ = +0.019 N) times the magnitude of the baseline thrust forces produced with the fins flapped in phase in -F configuration, 0.046 N (SD = ±0.006 N, *n* = 11).

#### 3.2.2. Root Mean Square Lateral Forces vs. Phase

The RMS lateral forces decreased consistently as the phase of the dorsal fin was increased or decreased from 0°. (Figure 5B,D). Maximum lateral forces were produced when fins were flapped in phase (0°) and had a value that was very close in magnitude to the value that was predicted when the total force was modeled as the sum of the forces produced by the individual fins. As the phase difference between the dorsal and/or anal fin and the caudal fin was increased, lateral forces decreased monotonically. As the phase difference between the fins neared ±180°, the lateral forces became very small, with a magnitude that was less than the RMS lateral forces produced by any individual fin (Figure 5B,D). 

A similar pattern of forces was produced by each of the three fin combinations tested; maximum lateral forces occurred when the fins were operated in-phase and the minimum forces occurred when the dorsal and anal fin led the caudal fin by ±180°. However, for each fin combination the range of lateral forces differed, both in their absolute value and when compared to the forces measured for the fin combination when operated in-phase. The dorsal and caudal fin combination (DC) produced the maximum lateral forces when flapped in phase (0°) and minimum lateral forces when flapped in opposition (180°). The minimum and maximum lateral force produced by the DC combination was 0.30 N (SD = ±0.015 N, *n* = 17) and 1.10 N (SD = ±0.011 N, *n* = 13), respectively, with the minimum being only 0.28 times the magnitude of in-phase lateral forces. 

The addition of the anal fin (DAC combination) increased the maximum lateral force but decreased the minimum lateral forces relative to the DC combination. The minimum and maximum lateral force produced by the DAC fin combination were 0.19 N (SD = ±0.017 N, *n* = 17) and 1.29 N (SD = ±0.014 N, *n* = 14), respectively, with the minimum being only 0.15 times the magnitude of in-phase lateral forces. Relative to the DC fin combination, the addition of the anal fin increased maximum lateral forces by 17% (0°) and decreased the minimum lateral force by 39% (180°). 

The addition of the flapping peduncle (PDAC combination) increased the lateral forces for all phases but slightly reduced the relative difference between the minimum and maximum lateral forces compared to other fin combinations (Figure 5B). The magnitude of PDAC in-phase lateral forces (0°) was 1.6 times larger than the lateral force produced by the DC combination. The minimum and maximum lateral forces produced with this fin combination was 0.62 N (SD = ±0.069 N, *n* = 12) and 1.79 N (SD = ±0.044 N, *n* = 16), respectively, with the minimum magnitude being 0.35 times the magnitude of the forces produced when the fins were flapped in phase. Relative to the DC fin combination, the PDAC fin combination increased both the magnitude of the maximum and minimum lateral force by 62% and 105%, respectively.

The relationship between RMS lateral forces and fin phase did not exhibit any phase shift when the position of the dorsal fin was moved to the forward position (Figure 5D). However, the range of lateral forces was smaller relative to the range of forces produced by the same fin combination with the dorsal fin was in the rearward position (-R). The minimum and maximum lateral force produced by the dorsal and caudal fin, with the dorsal in the forward position (DC-F), was 0.39 N (SD = ±0.011 N, *n* = 20) and 0.87 N (SD = ±0.028 N, *n* = 13), respectively, with the minimum being 0.45 of the magnitude of in-phase lateral forces (Figure 5D). Additionally, whereas minimum lateral forces were less than the forces produced by any individual fin with the dorsal fin in the rearward position (-R), in the dorsal forward configuration (-F) the minimum lateral forces were always greater than the lateral force produced by the dorsal fin alone.

### 3.3. Forces through Time

In addition to having significant effect on the mean thrust and RMS lateral forces, varying the phase of the fins caused considerable changes to the shape of the time-varying thrust and lateral forces. The pattern of change was similar for each of the three fin configurations. Pulses of thrust and drag during each fin beat were sharpest and had the greatest magnitude when the dorsal phase lead was near 0° and 180°, and became flatter and decreased in magnitude as the phase lead increased toward 90° and 270° (−90°) (Figure 5). In the following Section 3.3.1, details are provided for the dorsal and caudal configuration (DC) with subsequent sections describing the differences for the DAC and PDAC configurations and the forces from the 2D, thrust vs. lateral perspective (Section 3.3.2 and Section 3.3.3, respectively).

#### 3.3.1. Dorsal and Caudal Forces as Function of Time

##### Thrust Forces (DC-R Configuration)

**Figure 6:**
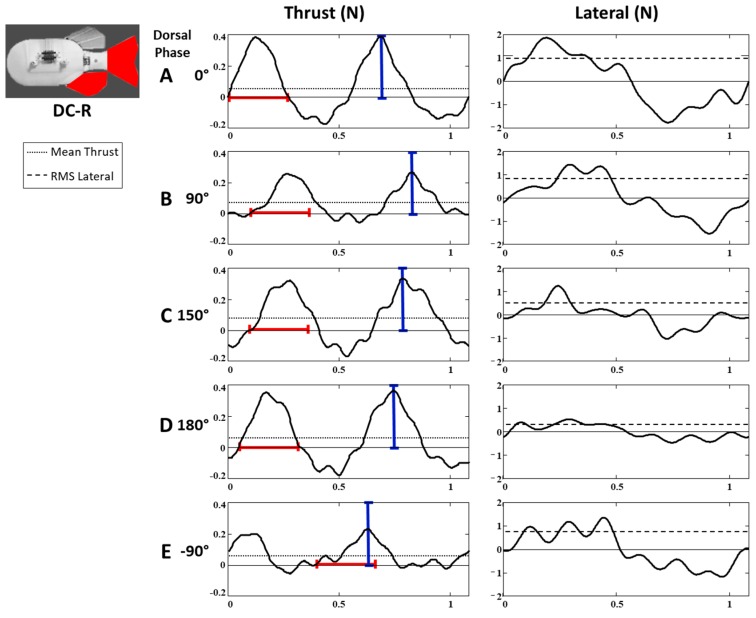
Relative to when (**A**) the dorsal and caudal fins are flapped in-phase, the shape of the thrust and lateral forces is noticeably different when the dorsal fin phase is (**B**) 90°, (**C**) 150°, (**D**) 180°, and (**E**) −90°. Bounded lines indicating the peak thrust magnitude (blue) and thrust pulse width (red) for the in-phase fins (**A**) are overlaid on force curves at all phases to aid in comparison.

When the dorsal and caudal fins were flapped in phase (ϴ = 0), the thrust forces (*T(t)*) had a similar shape to the forces produced by single fins, but with larger amplitudes (Figure 4A,B). During each half of the fin beat, the fins produced a steep, large amplitude pulse of thrust followed by a lower amplitude drag pulse (Figure 6A). The durations of the thrust and drag pulses were nearly equal, with thrust being produced during half of the fin beat (Figure 6A). 

**Figure 7:**
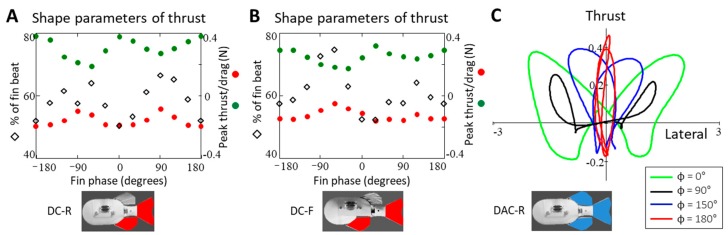
(**A**,**B**) The phase coordination of multiple fins greatly affects the magnitudes of peak thrust/drag produced and the proportion of the fin beat that produces thrust. (**C**) Two-dimensional forces can be manipulated from being a primarily lateral-dominant force to a thrust-dominant force.

As the phase lead of the dorsal fin was increased from 0° to 90°, the shape of the thrust pulse flattened, the magnitude of its peak decreased, and the proportion of the fin beat over which thrust was produced increased (Figure 6). Also, the magnitude of the peak drag force and the duration of the drag pulse decreased (Figure 7A). Collectively, these changes resulted in an increase in mean thrust over the fin beat as the phase difference between the dorsal and caudal fin increased, despite there being a general decrease in the magnitude of the thrust pulse. 

When the dorsal phase lead reached 90°, the magnitude of the peak of thrust was 70% of the magnitude when the fins were flapped in-phase, the duration of the thrust pulse increased to 68% of the fin beat, and the magnitude of the peak drag decreased to only 45% of its in-phase value (Figure 6B). Together, these changes resulted in a 20% increase in mean thrust relative to the fins when flapped in-phase (Figure 5A). 

As the phase lead of the dorsal fin was increased past 90° to 150°, the pattern of changes in the force curve reversed course. Rather than getting flatter and decreasing in magnitude, the thrust and drag pulses sharpened and increased in magnitude (Figure 6C). The duration of the thrust pulse also began to shorten (Figure 6A, Figure 7A). Despite these changes in shape being opposite to the changes that occurred when phase increased from 0° to 90°, the benefits to thrust outweighed the penalties in drag, and mean thrust continued to increase until reaching a maximum at a phase lead of 150°, a 41% increase relative to in-phase mean thrust. As the dorsal phase approached 180°, thrust and drag peaks continued to sharpen slightly and the relative durations of the fin beat that produced thrust and drag began to equalize. However, instead of mean thrust continuing to increase, the decreasing duration of the thrust pulse and increases in drag now overcome the increases in the thrust pulse magnitude resulting in a decrease in mean thrust. 

At 180° phase lead, the thrust curve had sharp, large amplitude thrust and drag pulses whose durations were approximately equal (Figure 7A), and overall shape closely resembled that which was produced by the fins when flapped in phase (Figure 6D). The peak magnitude of the thrust pulse was 2% greater than the in-phase magnitude and the peak magnitude of the drag pulse was nearly equal to that of the in-phase drag pulse (Figure 7A). The relative duration of the thrust pulse was 1% longer than the thrust pulse measured with the in-phase fins and, along with the slightly larger thrust pulse magnitude, may account for the slight increase mean thrust relative to the in-phase forces.

As dorsal phase increased from 180° to 270° (−90°), thrust and drag pulses again began to flatten and decrease in magnitude, in a similar fashion to the changes observed as phase increased from 0° to 90° (Figure 6E). However, the relative duration of the thrust and drag pulses remained nearly unchanged, and mean thrust decreased rather than increased, until it reaches a minimum at 270° (−90°). 

At dorsal phase lead of 270° (−90°), when mean thrust is at its minimum, the magnitudes of the peak of the thrust pulse was at its lowest; only 51% of the in-phase magnitude and the magnitude of the drag pulse was 45% of the in-phase drag. The relative duration of the fin beat during which thrust was produced was nearly unchanged (53%) (Figure 6A). Due in large part to the greater decrease in the thrust magnitude compared to drag; the mean thrust was 29% lower than was produced when the same fins were flapped in-phase.

As the dorsal phase lead was increased further from 270° to 360° (−90° to 0°), and fins were brought back into phase, thrust peaks increased in magnitude and sharpened, the magnitude of the drag pulse increased and the thrust-producing proportion of the fin beat increased until the shape and amplitude of the thrust forces matched previously measured in-phase forces (Figure 6A).

##### Lateral Forces (DC-R Configuration)

In contrast to the effect of fin phase on the thrust force shape, the change in the lateral force curves was largely in the magnitude of peak forces during each fin beat, and the general shape was not affected (Figure 6, right). When fins were flapped in phase, the shape of the lateral forces was very similar to that of the forces produced by a single fin, with large peaks of force that were balanced to each side, producing a near-zero mean. The magnitude of the peak lateral forces were largest when the fins were flapped in phase (ϴ = 0) and decreased monotonically as dorsal phase moved away from zero, independent of direction, until it reached a minimum when the dorsal phase was ±180°. At ±180°, the magnitude of the peak lateral forces was only 28% (Figure 6D, right) of the in-phase magnitude. 

#### 3.3.2. Effect of Additional Fins and Fin Configurations

The addition of the anal fin (DAC) amplified the previously described changes in peak magnitudes of thrust and drag as the phase lead of the dorsal and anal fins were varied relative to the caudal fin. For example, when dorsal and anal phase lead was 90°, thrust pulse duration was greatest (75%) and the thrust peak magnitude is 30% lower than the in-phase magnitude. Compared to the same conditions when the anal fin was not flapped (DC), this is an 8% increase in thrust pulse duration and a 3% greater decrease in the magnitude of peak thrust (Figure 7A). In the same way, when the phase leads of the dorsal and anal fin were 180° the lateral forces were closer to zero than the force produced by the DC combination under the same conditions.

Flapping all of the fins with the peduncle (PDAC) increased the overall magnitude of forces across the board. Although the previously mentioned changes in shape were generally the same, the shape of the forces produced by PDAC had larger thrust pulses, both in magnitude of the peaks as well as the relative proportion of the fin beat that produced thrust. When the dorsal and anal fins lead the peduncle and caudal fin by 90°, the shape of the thrust force exhibits the same flattening of the drag portion of the curve. In fact, this fin combination produces almost no drag at all between each thrust pulse, with the thrust pulse duration accounting for 77% of the fin beat and peak drag magnitude being only 38% of the in-phase magnitude. Compared to the same conditions for the DC configuration, this is a 9% increase in thrust pulse duration and a 6% decrease in the magnitude of peak drag.

When the dorsal fin was in the forward position and flapped with caudal fin (DC-F), changes in the shape of lateral forces with varying phase were the similar to those described for the dorsal-rearward configuration (-R). The changes in the sharpness/flatness of the thrust pulse described in Section 3.3.1. were the same. However, the effect of the phase on the magnitude of peak thrust was shifted in phase; similar to the manner in which the mean thrust vs. phase relationship changed when the dorsal fin was moved (Figure 7B). 

#### 3.3.3. The 2D Forces

Because the shape of the thrust and lateral forces changed simultaneously with phase, it can be useful to characterize their relative contribution to net forces on the body. During a fin beat, a single fin produced a 2D force vector whose shape is nearly symmetrical across the thrust axis and in which the lateral forces dominate the net force through time. The 2D force vector produced by the DAC combination of fins, when flapped in-phase, produced a very similar shape (Figure 7C).

As the dorsal phase lead increases from 0° and lateral forces decrease, 2D forces become more thrust-dominant, the shape of the 2D forces align with the thrust axis. At 180° phase lead, the shape of the 2D forces is nearly parallel with the thrust axis. Further increasing the dorsal phase lead, the lateral forces become more prominent and 2D force shape broadens along the lateral axis as the fins move back into phase.

### 3.4. Wake Flows

#### 3.4.1. Biorobotic Flow

Digital particle image velocimetry analysis was conducted for fin phase relationships that correspond to the baseline (in-phase), maximum (+150°) and minimum (−90°) mean thrust forces. By analyzing the flow at these extreme cases of thrust forces, changes in flow were easier to identify and characterize. A more detailed analysis of the wake flow interaction was conducted with the 2D simulated flow, and is discussed in Section 3.4.2.

**Figure 8:**
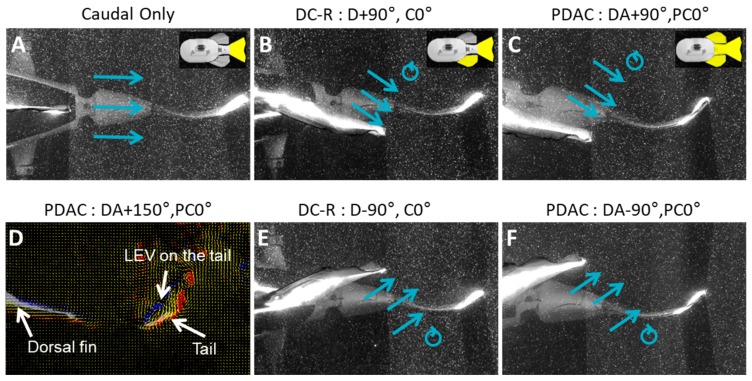
Particle image velocimetry images of the wake shed by dorsal fin and its flow over peduncle and caudal fin (large blue arrows)**.** (**A**) Flow encountered by the peduncle and caudal fin is greatly modified by the dorsal and anal fins, and differs significantly from the free stream. (**B**,**C**) When the dorsal fin leads the caudal fin by 90°, the angle of attack of the dorsal shed wake relative to the caudal fin is relatively small. (**E**,**F**) In contrast, the angle of attack of the dorsal shed wake relative to the caudal fin is much larger when the dorsal fin lags the caudal by 90°. (**D**) Appropriate timing of the dorsal and anal fins relative to the peduncle and caudal fin aligns the shed wake with the caudal fin encouraging the production of a strong leading-edge vortex (LEV), with velocity vectors (yellow arrows) and vorticity plotted in blue and red.

The dorsal/anal shed wake that is encountered by the caudal fin is very different than the flow that the caudal fin experiences when the dorsal and anal fins are stationary. With the dorsal and anal fins stationary, the flow that they shed remains in-line with the body. The angle at which the caudal fin engages with this flow changes almost exclusively due to the rotation of the caudal fin as it flaps (Figure 8A). When the dorsal and anal fins are flapped, vortices are produced and some of the flow is redirected laterally. As the dorsal fin changes direction at the most lateral point of the fin beat, a vortex is shed downstream. As the dorsal fin moves across the midline towards the opposite side of the body, it produced a wash of fluid that is ejected at an angle to the body. Once at the opposite point of its fin flap, the dorsal fin again changes direction and sheds another vortex with a rotation opposite of the previous vortex. As the dorsal fin returns to its starting position, another wash of fluid is ejected to the opposite side of body than the previous wash. This series of vortices and washes travel downstream and are encountered by the caudal fin.

The manner in which the caudal fin engages with the wake shed by the dorsal/anal fin is significantly impacted by the phase relationship between the fins. Depending on the phase lead of the dorsal and anal fins, these wakes can align with the leading edge of the caudal fin and cause there to be smooth flow over the curved caudal fin (Figure 8B,C); or the wake can approach the caudal fin at a large angle of attack, resulting in the flow accelerating as it changes direction and detaching from the caudal fin (Figure 8E,F). When maximum mean thrust is observed (+150° dorsal phase), the caudal fin is well aligned with the flow throughout much of the fin beat, its angle of attack remains small and a strong leading-edge vortex develops along the forward face of the fin (Figure 8D). In contrast, when the lowest mean thrust is observed (−90° dorsal phase), during most of the fin beat, the flow angle of attack relative to the caudal fin is large (≥40°), separation occurs on the aft face of the fin, and no leading-edge vortex is visible (Figure 8E,F). In addition to a large caudal angle of attack and flow separation under this phase condition, the timing of the caudal fin is such that its distal tip strikes the vortex produced by the upstream fin, disrupting its rotation. 

#### 3.4.2. Simulated Flow

**Figure 9:**
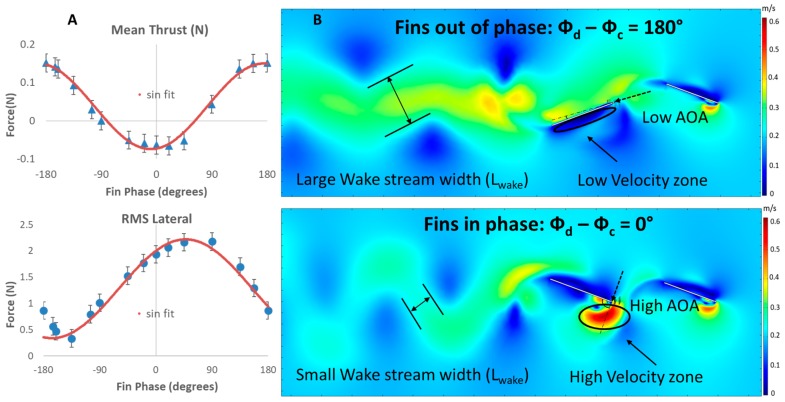
(**A**) Mean thrust and root mean square (RMS) lateral forces produced by simulated dorsal and caudal fins as the phase difference between the fins is varied. (**B**) Numerical simulations of flow when fins are out-of-phase (180°) vs. in-phase (0°). Appropriate timing of the caudal fin relative to the dorsal fin leads to a lower angle of attack (AOA) experienced by the caudal fin with a faster and larger wake stream that persists farther downstream.

Despite the differences in experimentation and numerical simulations, the relationship of fin forces versus phase calculated from the 2D numerical simulations was very similar to the relationships observed experimentally. The mean thrust forces and the lateral forces varied cyclically as the phase between the fins was varied. Flapping the dorsal and caudal fins in-phase produced minimum mean thrust of −0.51 N and produced maximum mean thrust of 1.21 N when flapped out-of-phase at +180° (Figure 9A). The RMS lateral forces decreased as the phase of the dorsal fin was either increased or decreased from 0°. The lateral forces were near maximum when the fins were flapped in-phase and were near minimum when the phase difference between the two fins was 180°.

The phase difference between the dorsal fin and the caudal fin altered the angle of attack of the caudal fin and created low/high velocity domain of fluid around the caudal fin throughout a fin beat. When maximum mean thrust and low lateral forces were observed (+180° dorsal phase; Figure 9A), the angle of attack of the caudal fin was relatively low throughout the fin beat. The range of the angle of attack of the caudal fin was from −25° to 35°, with near zero angle of attack when the caudal fin had reached its most lateral position. At caudal fin’s most extreme lateral position, a low velocity domain formed on the surface of the upstream facing side of the fin. This low velocity domain eventually accelerated and shed downstream during every fin beat (Figure 9B). In contrast, when the lowest mean thrust and near maximum lateral forces were observed (0° dorsal phase; Figure 9A), the angle of attack of the caudal fin was relatively high throughout the fin beat. The range of the angle of attack of the caudal fin was from −100° to 100°, with the peak value measured when the caudal fin was near its most lateral position. The wake shed by the dorsal fin interacted with the leading edge of the caudal fin, and a domain of high velocity fluid formed at the leading edge of the caudal fin (Figure 9B). This domain detached from the leading edge of the caudal fin and interacted with the surrounding fluid with stagnation points near the middle section of the caudal fin.

The phase relationship between the dorsal fin and the caudal fin affected the velocity and wake stream width of the downstream wake of the caudal fin. At +180° dorsal phase lead, a domain of high velocity fluid formed at the trailing edge of the caudal fin when the fin flapped from its most lateral position past the midline to the most lateral position on the opposite side. The velocity of the downstream wake shed by the caudal fin was approximately 75% faster than the free stream flow. This shed wake was smooth, continuous and focused around the midline of the dorsal and caudal fin with a big wake stream width. On the contrary, at 0° dorsal phase, the domain of high velocity fluid that formed at the trailing edge of the caudal fin decreased in size significantly as compared to the +180° dorsal phase lead case during a fin flap. The velocity of the downstream wake shed by the caudal fin was slower, now only 25% faster than the free stream flow. This shed wake was disjointed and concentrated farther away from the midline of the dorsal and caudal fins with a significantly smaller wake stream width.

## 4. Discussion

### 4.1. Forces

Propulsive forces were highly dependent on the phase relationships and the geometric locations of the peduncle, and the dorsal, anal and caudal fins. The effect is considerable: appropriate phase relations can more than double mean thrust or reduce lateral forces to nearly zero. This impact was similar for all fin combinations tested and the effect was increased with the use of additional fins or the peduncle. The changes in propulsive forces observed for the PDAC robot swimming in flow are representative of fin wake interactions more broadly and applicable to free-swimming robotic systems [44]. Also, of note is that these changes in mean forces occurred despite the dorsal and anal fins producing near-zero net thrust. This is not that unusual from a biological perspective, as some fish median fins also produce near-zero net thrust [5,48].

The variation of thrust with phase was more sensitive to changes in fin location than the variation of lateral forces with phase. Regardless of the dorsal/anal fin location along the body, the maximum lateral forces were produced when the fins were flapped in-phase and lateral forces were minimized when the fins were flapped with opposite phases (ϴ = ±180°). In contrast, changing the rostrocaudal distance between the fins greatly affected the relationship between the thrust force and phase, effectively shifting the phase at which maximum and minimum mean thrust occur. Because variation in fin location predominately affects the thrust–phase relationship, it is likely that a pair of fins with the appropriate geometric arrangement and phase relationship could produce maximum thrust and minimum lateral forces simultaneously.

In addition to the changes in mean forces, the impact of phase time-varying shape of the 2D forces produced during flapping varies significantly with phase as well. This variation in shape is patterned and certain shape changes are more affected by the changes in fin locations than others. This allows for the shaping of fin forces to meet desired force requirements including maximizing peak thrust, minimizing the drag produced during fin beats or achieving a desired thrust-to-lateral force ratio. For example, the maximum magnitude of peak thrust occurs when the fins are flapped in-phase (ϴ = 0°) or opposite phase (ϴ = ±180°), and is not affected by changes in geometry. Similarly, the relationship between the phase and the time-varying shape of the lateral forces is unaffected by a change in fin configuration with the maximum peak lateral magnitude always occurring when the fins are in-phase and decreasing as the fins move out of phase. In contrast, the relationship between phase and thrust pulse duration did change when the dorsal fin was relocated. It is through the combination of these shape changes and the different sensitivities of certain aspects of these shape changes to phase and geometry that give rise to the changes in mean forces.

**Figure 10:**
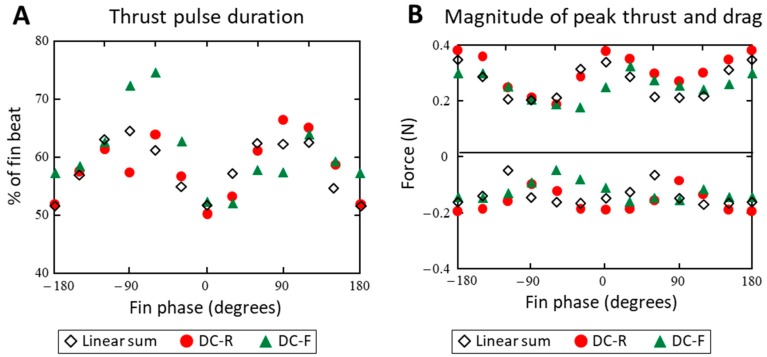
The variation in the time-dependent thrust forces differs from the linear sum of individual fin forces. (**A**) The thrust pulse duration for phases where thrust is maximized/minimized is least well accounted for. (**B**) Similar observations are found when comparing magnitudes of peak thrust and drag during a fin beat.

The observed changes in propulsive forces with phase and geometry are different from the net forces that would be expected if they were the result of the linear superposition of individual fin forces. For example, a linear model predicts no change to the mean thrust forces as the relative phase between fins was varied, but this investigation found that mean thrust forces can more than double with appropriate phasing between fins. A linear superposition model also does not account for the full extent of the variation in force shape with phase. In particular, the linear sum of individual fin forces would does not account for the changes in thrust pulse duration that is observed for the phase at which maximum or minimum thrust is produced (ϴ = 150° and −90, respectively) (Figure 10).

### 4.2. Flows

The fin phase and geometric relationships that produce large mean thrust correspond to flow conditions where the wake produced by upstream fin(s) smoothly transitions to, and is accelerated by, the downstream fin. This is observed in both the biorobotic experiments and the numerical simulations. Despite the fact that the wake around the dorsal fin was similar across all simulations, the caudal fin experienced a distinct wake change every time the phase relationship between fins was varied. When mean thrust forces are high, the caudal fin maintains a preferential angle of attack relative to the dorsal fin wake that reduces the disruption of the flow and produces a caudal fin wake that has a big wake stream width with larger momentum which enables the wake to remain coherent many fin lengths downstream (Figure 9B). In contrast, when thrust forces were low, the caudal fin angle of attack was larger and flow surrounding the caudal fin was ejected laterally, resulting in a wake that had a small wake stream width with smaller momentum which caused the wake to separate within three fin lengths downstream (Figure 9B). At every phase difference measurement except 180° phase lead, the dorsal fin wake interacts with the leading edge of the caudal fin and creates high velocity zones near the leading edge of the caudal fin. These large changes in velocity as flow moves along the caudal fin, including stagnation points near the middle portion of the fin, perturb the downstream wake and may be the cause of reduced thrust. By conditioning the flow to smoothly transition to and align with the downstream fin, appropriate phasing of the upstream fin may serve a similar purpose as inlet guide vanes in jet engines.

The flexibility and the interaction of the wake by upstream fin(s) also change how forces are affected by the phase and geometric relationships between the fins. For the PDAC robot, forces are a result of the complex, 3D interaction between a time-varying wake shed by the flexible dorsal/anal fin and only a portion of the downstream, flexible caudal fin as it is cupped and flapped. Using both the dorsal and anal fins doubles the area of the caudal fin that interacts with a modified flow, i.e., the wake overlap, which likely contributes to the increased effect the phase variation has on thrust and lateral forces. Flow visualizations recorded with the PDAC robot indicate that fin phase relationships that produce the high mean thrust exhibit a strong thrust-producing leading-edge vortex on the caudal fin due to the enhanced low-pressure zone on upstream face of the curved caudal fin (Figure 8D). In contrast, numerical simulations show that the wake interaction of rigid fins exhibits similar effects of fin phase on mean thrust and lateral forces without the associated vortex interaction or fin bending. This suggests that neither vortex enhancement nor fin flexibility is necessary to produce a large change in the magnitude of propulsive forces, a conclusion also found by the simulation study of Akhtar et al. [31].

### 4.3. Body Motion

By manipulating the full-range of phase and geometric relationships between the fins, net forces can be tailored to fulfil desired body motion goals. For a given pair of interacting fins, manipulating the phase relationship between them can drastically affect the magnitude and shape of the thrust and lateral forces. This would have a commensurate effect on the motion of the body. To maximize distance covered, a phase relationship can be chosen that maximizes mean thrust. If excessive lateral swaying of the body is undesirable, the phase relationship between the fins can be tuned to control or minimize the produced lateral forces. The tailoring of the time-varying forces within a fin beat can also be used to affect the desired body motion. When a very short burst of high acceleration is required during a single fin flap, all fins can move in phase to produce a large, short duration thrust and lateral force impulse that will alter the body trajectory. Additionally, due to the phase vs. lateral force relationship being relatively unaffected by changes in the distance between the fins, it may be possible to tune the fin geometric relationship such that a certain phase lag/lead between the fins produces the desired thrust and lateral forces simultaneously. In fact, preliminary investigations using a free-swimming multifinned robot, that executes similar fin kinematics as the PDAC robot, indicate that its body motions are consistent with what would be expected as a result of reported PDAC thrust and lateral forces under the same fin phase and geometric conditions.

The choices made when manipulating the shape of fin forces will vary depending on the dynamics of the body and desired motion requirements. The bodies of the model organisms used in this investigation have a shape that is robust to lateral and yaw perturbations, and more susceptible in pitch and roll. Force shaping with the fins can take into account this predisposition in body dynamics by placing greater importance on the optimization of thrust forces, especially in situations where manipulation of the fins’ geometric relationship is limited. Additionally, this study suggests that, if appropriately located along the body, the dorsal and anal fins may also play a role in counteracting the lateral force and yaw moment produced by the caudal fin, producing a lower net lateral acceleration of the body, and possibly increasing propulsive efficiency.

## 5. Conclusions

The relative phase and location of the peduncle and the dorsal, anal and caudal fins greatly affect the magnitude and shape of the produced thrust and lateral forces. Relative to flapping fins in-phase, appropriate choice of phase relations can more than double mean thrust; reduce lateral forces to nearly zero; or manipulate the shape of the thrust and drag producing portions of net forces. The forces that result from interacting fins are very different from the vector sum of forces from combinations of noninteracting fins. The changes in net forces are due, in large part, to time-varying wakes from dorsal and anal fins altering the flow experienced by the downstream body and caudal fin. When maximum mean thrust is produced, the phase relationship between the fins is such that the caudal fin maintains a preferential angle of attack relative to the wake produced by the upstream fin(s). This condition supports smooth flow over the surface of the caudal fin, enhancing the energy in the caudal fin wake in the direction of thrust. By manipulating phase and geometric relationships between the fins, net forces can be tailored to fulfil desired body motion goals, such as to reduce lateral body swaying or maximize distance traveled. This investigation has revealed for the first time that the coordination of multiple interacting fins enables mean and time-dependent forces to be shaped and modulated; a potentially powerful means of affecting the swimming forces produced by multifinned robotic systems.

The PDAC robotic model provides a realistic multifin platform for investigating the diversity of fish fin shapes, positions, kinematics, and the effect of altering fins on locomotor dynamics. Such studies are difficult, at best, to perform on living fishes. To better understand this fin–fin interaction and before it can be fully exploited in an engineered system, further studies are necessary to determine this fin–fin interaction is affected by changes in swimming speed, fin flapping frequency, wake overlap and fin flexibility.

## Figures and Tables

**Table 1:** Dimensions of the PDAC robot and support structure.

PDAC Fish Body				Overall PDAC Robot		
Length (mm)	Width (mm)	Height (mm)	Dorsal Fin Translation (mm)	Length (mm)	Width (mm)	Height (mm)
470	100	225	100	470	235	600

**Table 2:** Baseline, maximum and minimum mean thrust ($\bar{T}$) and the change in magnitude ($\Delta \bar{T}$) relative to in-phase forces measured for all four tested fin combinations/configurations.

	DC-R		DAC-R		PDAC-R		DC-F	
DA Phase	$\bar{T}$ (N)	$$\Delta \bar{T}(N)$$	$\bar{T}$ (N)	$$\Delta \bar{T}(N)$$	$\bar{T}$ (N)	$$\Delta \bar{T}(N)$$	$\bar{T}$ (N)	$$\Delta \bar{T}(N)$$
**In-phase (0°)**	0.052	0.000	0.039	0.000	0.108	0.000	0.046	0.000
**Max (150°)**	0.077	+0.025	0.087	+0.048	0.146	+0.038	0.065	+0.019
**Min (−90°)**	0.035	−0.016	0.020	−0.019	0.076	−0.032	0.034	−0.012
**Δ (min − max)**	-	0.041	-	0.067	-	0.070	-	0.031

DAC-R: Dorsal, anal and caudal fin forces, in dorsal-rearward configuration; DC-F: Dorsal and caudal fin forces, in dorsal-forward configuration; DC-R: Dorsal and caudal fin forces, in dorsal-rearward configuration; PDAC-R: Peduncle, dorsal, anal and caudal fin forces, in dorsal-rearward configuration.
